# Effects of Whey Protein Supplementation on Body Composition, Muscular Strength, and Cardiometabolic Health in Older Adults: A Systematic Review with Pairwise Meta-Analysis

**DOI:** 10.3390/healthcare13212814

**Published:** 2025-11-05

**Authors:** Mousa Khalafi, Saeid Fatolahi, Reihaneh Jafari, Sara K. Rosenkranz, Michael E. Symonds, Zeinab Abbaszadeh Bidgoli, Maria Luz Fernandez, Farnaz Dinizadeh, Alexios Batrakoulis

**Affiliations:** 1 Department of Sport Sciences, Faculty of Humanities, University of Kashan, Kashan 87317-53153, Iran; reihawnehjafari@gmail.com (R.J.); abbaszadezeinab1380@gmail.com (Z.A.B.); 2 Department of Physical Education and Sport Sciences, Faculty of Humanities, Tarbiat Modares University, Tehran 111-14115, Iran; saeed.ft1370@gmail.com; 3 Department of Kinesiology and Nutrition Sciences, University of Nevada Las Vegas, Las Vegas, NV 89154, USA; sara.rosenkranz@unlv.edu; 4 Centre for Perinatal Research, Academic Unit of Population and Lifespan Sciences, School of Medicine, University of Nottingham, Nottingham NG7 2UH, UK; michael.symonds@nottingham.ac.uk; 5 School of Nutrition and Wellness, University of Arizona, Tucson, AZ 85712, USA; maria-luz.fernandez@uconn.edu; 6 Department of Sport Sciences, Tabriz Branch, Azad University, Tabriz 51579-44533, Iran; farnazdinizadeh@gmail.com; 7 Department of Physical Education and Sport Science, Democritus University of Thrace, 769100 Komotini, Greece; 8 Department of Physical Education and Sport Science, University of Thessaly, 42100 Trikala, Greece

**Keywords:** whey protein, cardiometabolic health, body composition, glycemic control, lipid profile, muscular strength, older adults

## Abstract

**Background/Objectives:** Whey protein (WP) can play a role in improving muscle mass and function. However, the effects of WP supplementation on cardiometabolic health parameters such as fasting blood glucose, insulin, and homeostatic model assessment of insulin resistance (HOMA-IR), fasting triglyceride, total cholesterol, low-density lipo-protein cholesterol, and high-density lipoprotein cholesterol have not been fully elucidated in older adults and are therefore the aim of the present systematic review and pairwise meta-analysis. **Methods:** A comprehensive search of major databases (PubMed, Web of Science, and Scopus) was conducted up to January 2025 for English-language randomized controlled trials examining WP supplementation, either alone or in combination with resistance training in older adults. Eligible studies reported at least one of the following outcomes: body fat mass, body fat percentage, lean body mass, waist circumference, waist-to-hip ratio, muscular strength, glycemic parameters, and lipid profiles. **Results:** A total of 25 studies involving 1454 participants with mean ages ranging from 64 to 84 years, with body mass indexes ranging from 21 to 31 kg·m^2^ were included, from an initial 868 records identified through database searches. Overall, compared with controls, WP supplementation increased lower-body muscular strength [SMD: 0.16 (95% CI: 0.04 to 0.28), *p* = 0.007; 19 trials], but without significantly changing upper-body muscular strength, body composition, or other cardiometabolic health markers. However, WP supplementation increased fasting insulin and homeostatic model assessment of insulin resistance. Subgroup analyses showed that whey protein plus resistance training increased lean body mass, while WP alone improved lower-body strength, with no other significant effects observed. **Conclusions:** WP supplementation moderately increases lower-body muscle strength in older adults. However, it does not show any significant benefits for body composition or cardiometabolic health markers. Conversely, increased fasting insulin and HOMA-IR were documented. These findings emphasize the need for careful examination of the metabolic effects of WP supplementation in future longer-term trials.

## 1. Introduction

The global middle-aged and older adult population continues to increase, emphasizing the importance of age-related diseases as public health challenges [1,2] particularly given that the number of people older than 60 years is expected to double by 2050. The main age-associated issues are progressive the loss of skeletal muscle mass, strength, and function, termed sarcopenia, and associated redistribution of fat mass with increased intra-abdominal fat [3]. Such changes lead to the development of metabolic and cardiovascular diseases [4,5,6]. In addition, age-related cardiometabolic diseases are associated with other conditions that are known to increase in prevalence with aging. These conditions include insulin resistance, dyslipidemia, hypertension, and chronic low-grade inflammation [7,8,9,10,11,12,13].

As individuals age, they may encounter difficulties in preserving muscle mass compared to younger adults. This is due to a decline in the body’s capacity to synthesize and regenerate muscle tissue, which is a process that requires adequate protein intake [14]. Furthermore, older adults may experience a heightened rate of muscle protein breakdown, which can exacerbate muscle loss [15]. Recent findings indicate that the protein requirements of older adults may exceed those of the general adult population to achieve a comparable muscle-building response [16]. The evidence suggests that the aforementioned factors, when considered in conjunction with the demonstrated tendency of older adults to exhibit diminished appetite and reduced food intake, may indicate a potential benefit from the administration of nutritional supplements in this demographic [17]. A range of associated concerns have been identified, including but not limited to dental health issues and swallowing problems [18]. Collectively, these factors indicate that older adults necessitate higher protein intakes compared to younger adults [19].

Adequate nutrient intake, especially adequate protein meeting the recommended daily intake of 1.0 to 1.2 g per kilogram of body weight [20], can prevent and/or delay the effects of aging on muscle mass and function [21], with evidence indicating that protein supplementation can even improve lean body mass in older adults [21,22,23,24,25,26]. Whey protein (WP), derived from milk, is a high-quality source of protein, often used in protein supplements, containing bioactive peptides and branch chain amino acids which are rapidly digested and delivered to the small intestine intact [27,28]. WP increases postprandial amino acid availability and stimulates protein synthesis rates, and thus has the potential to ameliorate age-related loss of skeletal muscle mass and function [29,30]. Several meta-analyses have shown that (WP) supplementation may improve markers of sarcopenia in older adults, especially when combined with resistance training [31,32,33,34]. However, the effects of WP supplementation on cardiometabolic health in older adults has been a subject of considerable research, demonstrating improvements in several markers, including glycolipid metabolism [35,36]. These benefits are particularly pronounced when supplementation is combined with resistance training [36]. WP supplementation also reduces post-meal blood glucose peak values by enhancing insulin and gut hormone responses [37]. The long-term effects on glycemic control (glycated hemoglobin), blood pressure, and high-density lipoprotein cholesterol are uncertain [38,39]. A comprehensive review of the available literature suggests that WP is a safe and beneficial supplement for improving metabolic health in older adults with no underlying kidney issues. In general, some evidence suggests the potential for improvements in f insulin resistance, dyslipidemia, vascular function, blood pressure, and chronic low-grade inflammation [28,35,36,38,39].

Current meta-analyses suggest that WP has beneficial effects on some lipid profile markers and glycemic control in healthy adults (aged <50 years) with overweight or obesity, and in patients with metabolic syndrome and related conditions [40,41]. The effects of supplementation on body composition, muscle strength, and cardiometabolic health in older adults remains uncertain due to inconsistent findings, limited long-term data, and the inadequate control of baseline dietary protein. This research gap hinders evidence-based recommendations for preserving performance and health in aging populations. Consequently, the present systematic review and meta-analysis, employing a pair-wise approach, sought to elucidate these effects and provide novel data on the role of WP supplementation in critical health-related physiological indicators within this important demographic.

## 2. Materials and Methods

This systematic review and pairwise meta-analysis were conducted according to the Preferred Reporting Items for Systematic Reviews and Meta-Analyses (PRISMA) guidelines [42] and additional methods as presented in the Cochrane Handbook for Systematic Reviews of Interventions. The study protocol was prospectively registered in the International Prospective Register of Systematic Reviews (PROSPERO) with ID: CRD420251059088.

### 2.1. Search Strategy

A comprehensive search was conducted in three primary electronic databases including PubMed, Web of Science, and Scopus, to identify original English language studies investigating the effects of WP in older adults up to January 2025. When available in each database, filters including human, English languages, and article or document, were applied. In addition, the reference lists of included studies, previous meta-analyses, and Google Scholar were manually searched to identify any further eligible studies. The searches were conducted by two independent authors (M.Kh. and R.J.). The search strategy is shown in Supplementary Table S1.

### 2.2. Study Selection and Eligibility Criteria

Studies were considered eligible for inclusion if they were peer-reviewed and met the following criteria based on the PICOS framework: Population: study of human participants with mean ages ≥60 years, regardless of biological sex or health status; Intervention: intervention included receiving WP supplements for at least 2 weeks; Comparator: control groups receiving either a placebo/control or carbohydrate supplement (PLA/CON); Outcomes: changes in body composition [body fat mass, body fat percentage (BF%), lean body mass (LBM), waist circumference (WC), or waist-to-hip ratio (WHR)]; glycemic markers [fasting glucose, fasting insulin, or Homeostatic Model Assessment for Insulin Resistance (HOMA-IR); fasting lipid profiles such as triglycerides (TGs), total cholesterol (TCH), low-density lipoprotein cholesterol (LDL), or high-density lipoprotein cholesterol (HDL); upper and lower-body muscular strength; Study design: randomized controlled trials with parallel or crossover designs. In addition, if studies included combined WP with exercise training versus exercise training alone, they were included. Also, studies that included WP plus calcium were included. Exclusion criteria were as follows: non-original studies, non-English language studies, and studies that included WP with co-supplementation, such as vitamin D, to isolate the protein-specific effects and avoid confounding from vitamin D metabolism. Study selection was conducted by three independent authors (R.J., F.D., and Z.A.B.) and any disagreements were resolved by discussion with other authors (S.F. and M.Kh.). All retrieved studies were imported into EndNote (version 21), and after removing duplicate records, the titles and abstracts of all remaining studies were screened. Then, the full texts of the remaining eligible studies were screened to determine the final studies included in the systematic review and pairwise meta-analysis.

### 2.3. Data Extraction and Synthesis

The following information was extracted from each included study: (1) first author name and publication year, study design, and sample size; (2) participant characteristics including age, biological sex, BMI, and health status; (3) WP supplement characteristics including type, dosage, and time of consumption; (4) exercise training characteristics including mode, intensity, duration, and protocol; and (5) outcome variables measured. In addition, to perform analyses, the following data were extracted: mean changes (post values—pre values) and their related standard deviation (SD) and sample size, or mean and SD from pre- and post-intervention values to calculate mean and SD changes using the relevant formula as recommended by the Cochrane handbook. In addition, when required, these data were extracted from figures using Getdata Graph Digitizer software (2.26), or were calculated from other data such as medians and interquartile ranges (IQRs) using the relevant formulas [43,44,45]. However, when required, the corresponding authors were contacted to request missing data. Three independent authors (R.J., F.D. and Z.A.B.) extracted the data, and any disagreements were resolved by discussion with other authors (M.Kh. and S.F.).

### 2.4. Quality Assessment

In order to assess the overall quality of included studies, we used the Physiotherapy Evidence Database (PEDro) Scale, a valid measure of the methodological quality of clinical trials [46]. Assessment was undertaken across 11 domains and is summarized in Supplementary Table S2. Higher scores indicate higher quality studies. Two independent authors (R.J. and S.F.) evaluated risk of bias for each domain, and any disagreements were resolved by discussion with another author (M.Kh.) (Supplementary Table S2).

### 2.5. Statistical Analysis

To investigate the effects of WP against PLA/CON groups for each outcome, separate meta-analyses were conducted using Comprehensive Meta-Analysis version 3 (CMA3) software. Effect sizes were determined according to measurement method units, where we used weighted mean differences (WMDs) or standardized mean differences (SMDs) with 95% confidence intervals (CIs) when units were the same or different, respectively. Random effects models were used to calculate effect sizes and generate forest plots. Subgroup analyses were conducted based on WP with and without exercise training. Heterogeneity was assessed using I2 and Cochrane Q statistics, and I2 was interpreted as low (<25%), moderate (25% to <50%), and high (50% to <75%) heterogeneity [47]; whereas Q was significant if *p* < 0.05. Publication bias was assessed using the visual interpretation of funnel plots and significant Egger’s test results if *p* < 0.10. The trim and fill method was used to correct the effect sizes when there was publication bias based on the visual interpretation of funnel plots. To evaluate the stability of results, sensitivity analysis was performed by removing individual studies. In addition, subgroup analyses of WP supplementation with and without resistance training, were performed when at least three studies were available for each subgroup.

## 3. Results

### 3.1. Search Strategy

**Figure 1:**
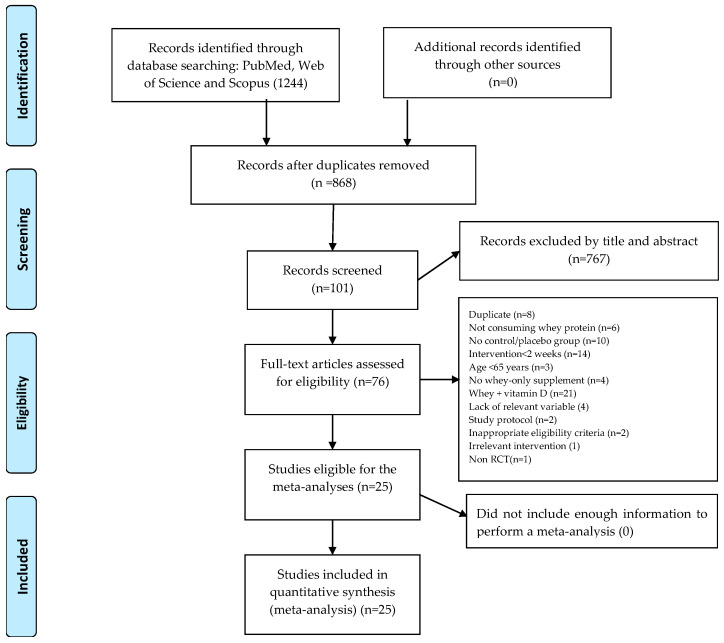
Flow diagram of systematic literature search.

The flow of the searches and study selection is summarized in Figure 1. Overall, 1244 records were found during the initial searches, of which 868 remained after removing duplicates. An additional 766 studies were excluded after the first step of screening (titles and abstracts), and subsequently 76 were excluded for the reasons summarized in Figure 1. Finally, 25 studies met all inclusion criteria and were included in the meta-analyses. All included studies were randomized controlled trials (RCTs), with 15 including WP plus exercise training versus exercise training alone groups [48,49,50,51,52,53,54,55,56,57,58,59,60,61,62], 7 comparing WP to a control group [63,64,65,66,67,68,69], and 3 including all of these study groups [36,70,71]. In addition, data were extracted manually from 3 articles [36,61,71].

### 3.2. Literature Characteristics and Quality Assessment

Included studies comprised 1454 participants with mean ages ranging from 64 to 84 years, and BMIs ranging from 21 to 31 kg.m^2^. The health status of participants varied from healthy to chronic diseases such type 2 diabetes. Intervention durations ranged from 8 weeks to 2 years, with 12 and 16 weeks used in a majority of studies. The dosage of WP supplementation varied from 15 to 35 g per day, with and without leucine enrichment. The PLA/CON groups received maltodextrin or non-protein supplements (see Table 1 and Table S2 for detailed intervention characteristics). According to PEDro scores, methodological quality was fair in 3 studies, good in 18 studies, and excellent in 4 studies (Supplementary Table S2).

### 3.3. Meta-Analysis

#### 3.3.1. Body Composition

**Figure 2:**
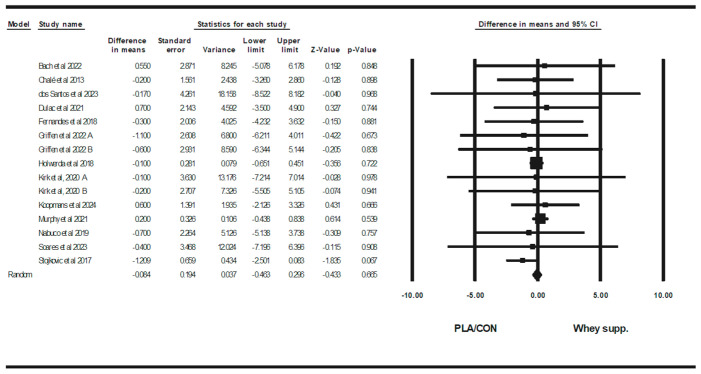
Forest plot of the effects of WP supplementation versus PLA/CON on body fat mass. Data are reported as WMD (95% confidence limits) [50,51,52,53,55,58,62,63,64,67,68,70,71]. Note. A and B are from the same study (Griffen et al., 2022 [70]; Kirk et al., 2020 [71]).

**Figure 3:**
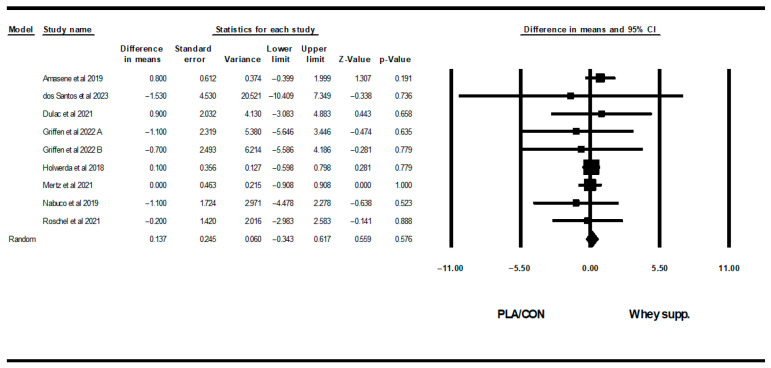
Forest plot of the effects of WP supplementation versus PLA/CON on body fat percentage (BF%). Differences in means are reported as WMD (95% confidence limits) [48,52,55,58,60,63,66,70]. Note. A and B are from the same study (Griffen et al., 2022 [70]).

**Figure 4:**
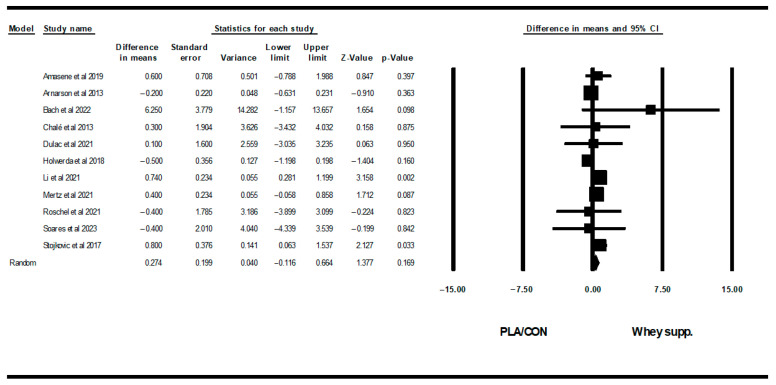
Forest plot of the effects of WP supplementation versus PLA/CON on lean body mass (LBM). Data are reported as WMD (95% confidence limits) [48,49,50,51,52,55,60,62,65,66,68].

**Figure 5:**
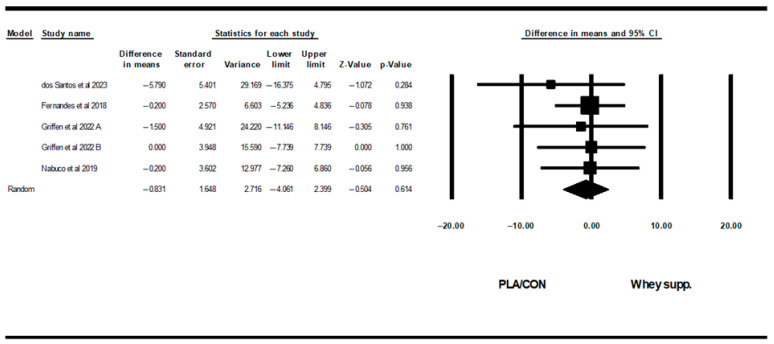
Forest plot of the effects of WP supplementation versus PLA/CON on waist circumference (WC). Data are reported as WMD (95% confidence limits) [53,58,63,70]. Note. A and B are from the same study (Griffen et al., 2022 [70]).

**Figure 6:**
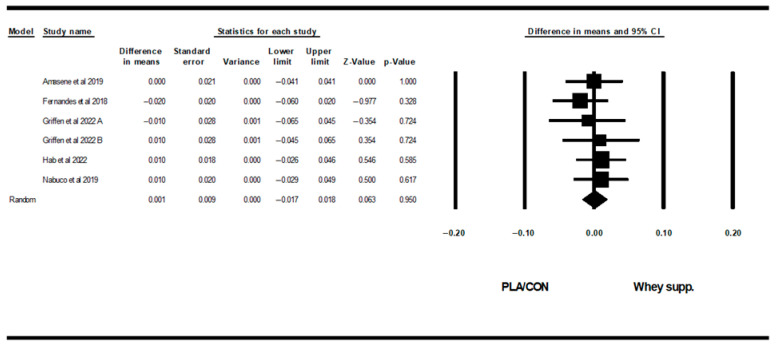
Forest plot of the effects of WP supplementation versus PLA/CON on waist-to-hip ratio (WHR). Data are reported as WMD (95% confidence limits) [48,53,54,58,70]. Note. A and B are from the same study (Griffen et al., 2022 [70]).

WP supplementation did not change fat mass [WMD: −0.08 kg (95% CI: −0.46 to 0.29 *p* = 0.66; 15 trials], BF% [WMD: 0.13% (95% CI: −0.34 to 0.61), *p* = 0.57; 9 trials], LBM [WMD: 0.27 kg (95% CI: −0.11 to 0.66), *p* = 0.16; 11 trials], WC [WMD: −0.83 cm (95% CI: −4.06 to 2.39 *p* = 0.61; 5 trials], or WHR [WMD: 0.00 cm (95% CI: −0.30 to 0.31 *p* = 0.96; 6 trials] significantly more than PLA/CON (Figure 2, Figure 3, Figure 4, Figure 5 and Figure 6).

Heterogeneity was not significant fat mass (I2 = 0.00, *p* = 0.99), BF% (I2 = 0.00, *p* = 0.96), WC (I2 = 0.00, *p* = 0.91), or WHR (I2 = 0.00, *p* = 0.88) and significant for LBM (I2 = 46.08, *p* = 0.04). Visual interpretation of funnel plots suggested publication bias, but Egger’s test results did not confirm this bias for fat mass (*p* = 0.56), BF% (*p* = 0.32), LBM (*p* = 0.72), or WC (*p* = 0.17). Both visual interpretation of funnel plots and Egger’s test results did not suggest publication bias for WHR (*p* = 0.76).

#### 3.3.2. Glycemic Markers

**Figure 7:**
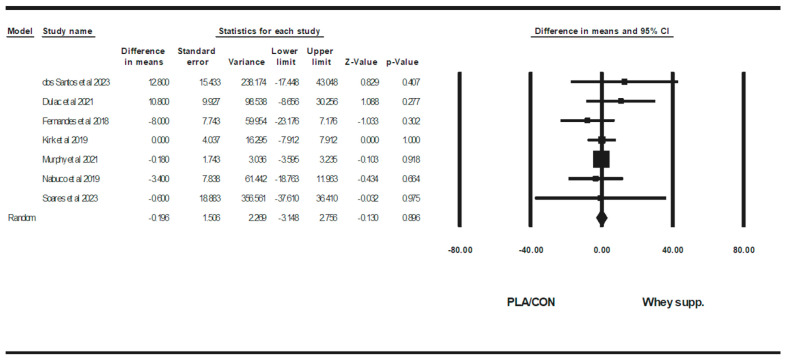
Forest plot of the effects of WP supplementation versus PLA/CON on fasting glucose. Data are reported as WMD (95% confidence limits). WMD: weighted mean difference [52,53,58,61,62,63,67].

**Figure 8:**
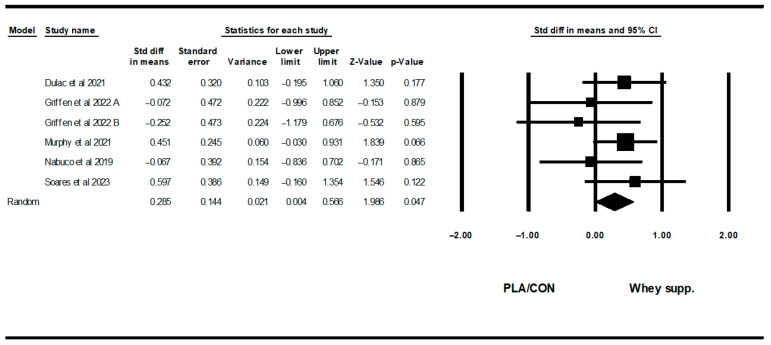
Forest plot of the effects of WP supplementation versus PLA/CON on fasting insulin. Data are reported as SMD (95% confidence limits) [52,58,62,67,70]. Note. A and B are from the same study (Griffen et al., 2022 [70]).

**Figure 9:**
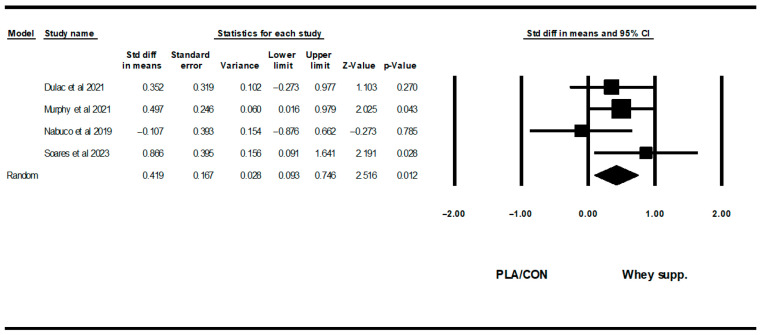
Forest plot of the effects of WP supplementation versus PLA/CON on HOMA-IR. Data are reported as SMD (95% confidence limits) [52,58,62,67].

WP supplementation did not change fasting glucose [WMD: −0.19 mg/dl (95% CI: −3.14 to 2.75), *p* = 0.89; 7 trials] significantly more than PLA/CON, but did significantly increase fasting insulin [SMD: 0.28 (95% CI: 0.00 to 0.56), *p* = 0.04, 6 trials] and HOMA-IR [SMD: 0.41 (95% CI: 0.09 to 0.74), *p* = 0.01; 4 trials] (Figure 7, Figure 8 and Figure 9).

Heterogeneity was not significant for fasting glucose (I2 = 0.00, *p* = 0.79), fasting insulin (I2 = 0.00, *p* = 0.55), or HOMA-IR (I2 = 6.89, *p* = 0.35). Visual interpretation of funnel plots suggested publication bias, but Egger’s test results did not confirm this bias for fasting glucose (*p* = 0.69) or fasting insulin (*p* = 0.10). Both visual interpretation of funnel plots and Egger’s test results did not suggest publication bias for HOMA-IR (*p* = 0.82).

#### 3.3.3. Lipid Profiles

**Figure 10:**
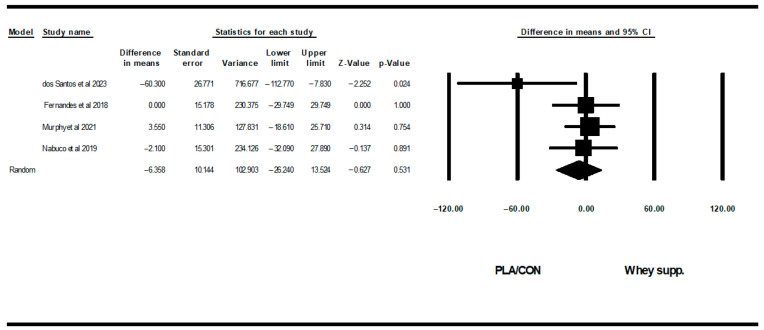
Forest plot of the effects of WP supplementation versus PLA/CON on TG. Data are reported as WMD (95% confidence limits) [53,58,63,67].

**Figure 11:**
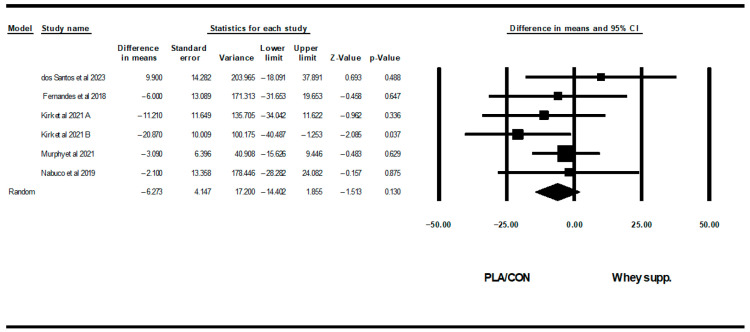
Forest plot of the effects of WP supplementation versus PLA/CON on TCH. Data are reported as WMD (95% confidence limits) [53,58,63,67,72]. Note. A and B are from the same study (Kirk et al., 2020 [71]).

**Figure 12:**
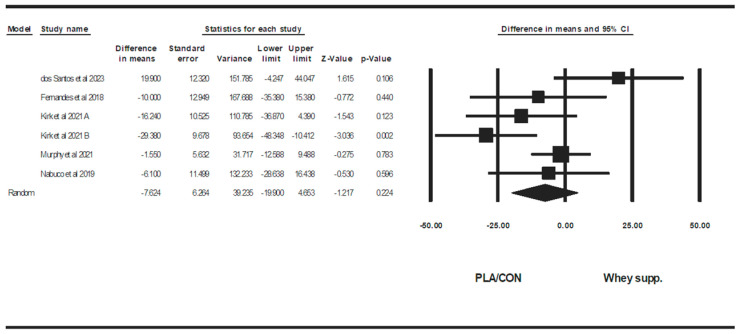
Forest plot of the effects of WP supplementation versus PLA/CON on LDL. Data are reported as WMD (95% confidence limits) [53,58,63,67,72]. Note. A and B are from the same study (Kirk et al., 2020 [71]).

**Figure 13:**
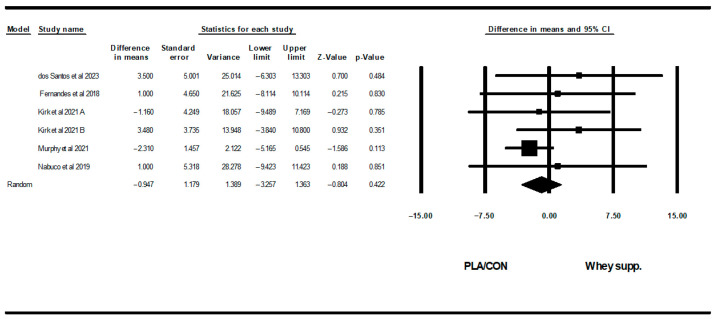
Forest plot of the effects of WP supplementation versus PLA/CON on HDL. Data are reported as WMD (95% confidence limits) [53,58,63,67,72]. Note. A and B are from the same study (Kirk et al., 2020 [71]).

WP supplementation did not change TG [WMD: −6.35 mg/dl (95% CI: −26.24 to 13.52), *p* = 0.53; 4 trials], TCH [WMD: −6.27 mg/dl (95% CI: −14.40 to 1.85), *p* = 0.13; 6 trials], LDL [WMD: −7.62 mg/dl (95% CI: −19.90 to 4.65), *p* = 0.22; 6 trials], or HDL [WMD: −0.94 mg/dl (95% CI: −3.25 to 1.36), *p* = 0.42; 6 trials] significantly more than PLA/CON (Figure 10, Figure 11, Figure 12 and Figure 13).

Heterogeneity was not significant for TG (I2 = 39.41, *p* = 0.17), TCH (I2 = 0.00, *p* = 0.55), or HDL (I2 = 0.00, *p* = 0.64), but was significant for LDL (I2 = 57.90, *p* = 0.03). Visual interpretation of funnel plots suggested publication bias, but Egger’s tests did not confirm this bias for TCH (*p* = 0.91) and TG (*p* = 0.06). Both visual interpretation of funnel plots and Egger’s test results suggested publication bias for HDL (*p* = 0.04), while both visual interpretation of funnel plots and Egger’s test results did not suggest publication bias for LDL (*p* = 0.81).

#### 3.3.4. Muscular Strength

**Figure 14:**
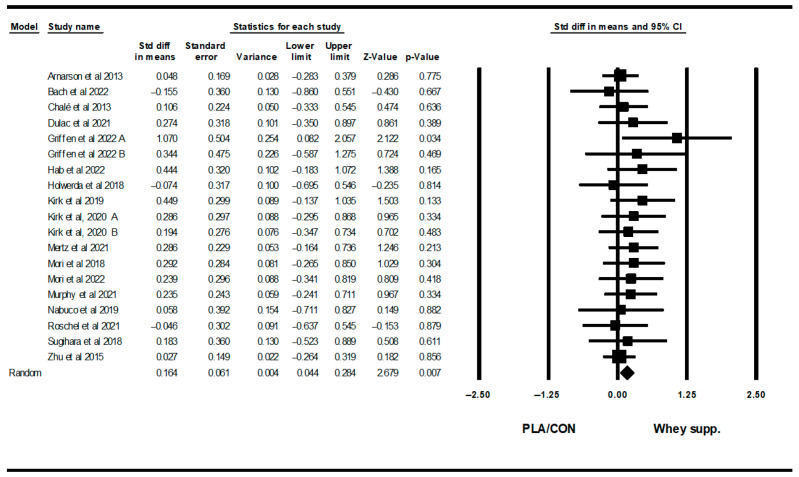
Forest plot of the effects of WP supplementation versus PLA/CON on upper-body muscular strength. Data are reported as SMD (95% confidence limits) [49,50,51,52,54,55,56,57,58,59,60,61,66,67,69,70,71]. Note. A and B are from the same study (Griffen et al., 2022 [70]; Kirk et al., 2020 [71]).

**Figure 15:**
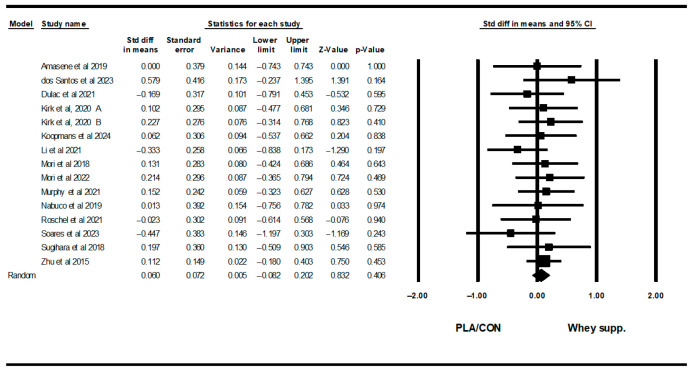
Forest plot of the effects of WP supplementation versus PLA/CON on lower-body muscular strength. Data are reported as SMD (95% confidence limits) [48,52,56,57,58,59,60,62,63,64,65,67,69,71]. Note. A and B are from the same study (Kirk et al., 2020 [71]).

WP supplementation did not change upper-body muscular strength [SMD: 0.06 (95% CI: −0.08 to 0.20), *p* = 0.40; 19 trials], but increased lower-body muscular strength [SMD: 0.16 (95% CI: 0.04 to 0.28), *p* = 0.007; 15 trials] significantly more than PLA/CON (Figure 14 and Figure 15).

Heterogeneity was not significant for upper-body muscular strength (I2 = 0.00, *p* = 0.91 or lower-body muscular strength (I2 = 0.00, *p* = 0.95). Visual interpretation of funnel plots suggested publication bias, but Egger’s tests did not confirm this bias for upper-body muscular strength (*p* = 0.76). In addition, both visual interpretation of funnel plots and Egger’s test results suggested publication bias for lower-body muscular strength (*p* = 0.03).

#### 3.3.5. Subgroup Analyses

Subgroup analyses showed that WP supplementation without resistance training did not change fat mass [WMD = −0.05 kg, (95% CI: −0.60 to 0.49), *p* = 0.84], BF% [WMD = −0.03%, (95% CI: −0.92 to 0.85), *p* = 0.93], TCH [WMD = −6.14 mg/dl (95% CI: −20.97 to 8.67), *p* = 0.41], LDL [WMD = −4.46 mg/dl (95% CI: −28.09 to 19.17), *p* = 0.71], HDL [WMD = −0.05 mg/dl (95% CI: −4.26 to 4.15), *p* = 0.98], upper-body muscular strength [SMD = 0.09 (95% CI: −0.10 to 0.28), *p* = 0.35] or lower-body muscular strength [SMD = 0.14 (95% CI: −0.04 to 0.34), *p* = 0.13] compared with PLA/CON. However, in the same subgroup analysis, LBM increased significantly [WMD = 0.60 kg (95% CI: 0.31 to 0.90), *p* = 0.00].

Also, WP supplementation is combined with resistance training, did not alter fat mass [WMD = −0.10 kg, (95% CI: −0.63 to 0.41), *p* = 0.68], BF% [WMD = 0.20%, (95% CI: −0.36 to 0.77), *p* = 0.47], LBM [WMD = −0.20 kg (95% CI: −0.55 to 0.14), *p* = 0.24], WC [WMD = −0.39 cm (95% CI: −4.17 to 3.37), *p* = 0.83], WHR [WMD = 0.00 (95% CI: −0.01 to 0.01), *p* = 0.96], fasting glucose [WMD = −0.75 mg/dl, (95% CI: −6.73 to 5.23), *p* = 0.80], fasting insulin [SMD = 0.27 (95% CI: −0.10 to 0.64), *p* = 0.15], TCH [WMD = −6.87 mg/dl (95% CI: −21.17 to 7.41), *p* = 0.34], LDL [WMD = −11.19 mg/dl (95% CI: −24.24 to 1.86), *p* = 0.09], HDL [WMD = 0.12 mg/dl (95% CI: −5.16 to 5.42), *p* = 0.96], upper-body muscular strength [SMD = 0.02 (95% CI: −0.19 to 0.23), *p* = 0.83], in comparison with PLA/CON. On the other hand, in this subgroup, lower-body muscular strength increased significantly [SMD = 0.17 (95% CI: 0.02 to 0.32), *p* = 0.02].

## 4. Discussion

To the best of our knowledge, this is the first meta-analysis of its kind to focus on older adults, to utilize pairwise analysis, and to examine the effects of WP supplementation alone versus WP plus exercise in this cohort. The primary conclusions of the present study indicated that WP supplementation did not result in substantial alterations in body composition or various cardiometabolic health-related indicators. However, it did enhance muscular strength in older adults. Specifically, WP had a small positive effect on lower body muscular strength in older adults, without affecting body composition (including LBM), glycemic markers, or lipid profiles. Future research must investigate the reported increases in fasting insulin and HOMA-IR to identify possible risks for this population. Subgroup analyses revealed that WP combined with resistance training increased lean body mass but showed no further benefits in other outcomes, whereas WP alone improved lower-body strength.

Generally, it has been documented that fat mass reduction and increased LBM are advantageous for elderly individuals who are most at risk for sarcopenia [72]. Collectively, it should be noted that the absence of improved body composition observed in the present systematic review would logically be associated with no changes in markers of cardiometabolic health [73], and is in accordance with an earlier meta-analysis [31]. However, in healthy, overweight/obese adults aged <50 years, WP supplementation enhanced body fat loss compared to participants who were normal weight [41]. Further studies investigating WP supplementation in older adults with or without obesity are thus warranted. In addition, the present review corroborates the notion that a combination of WP supplementation and resistance training may hold particular significance, as extant evidence indicates a favorable impact on LBM among elderly individuals [74].

WP is characterized by its high content of essential amino acids, particularly branched-chain amino acids, that modulate insulin secretion [75] and exhibit a positive effect on pancreatic beta-cell function [76]. The present meta-analysis did not reveal beneficial effects of WP on glycemic markers despite some evidence that it can enhance glucose homeostasis, and prevent insulin resistance [77]. In fact, the current analysis suggested that there may be detrimental effects on fasting insulin levels in older adults, though only six studies were included for that outcome. We also found no improvements in lipid homeostasis, thereby contrasting with previous rodent studies using WP supplementation that indicated a cholesterol lowering effect mediated by downregulating hepatic cholesterol synthesis and reducing the production of its precursors [78]. Conversely, a meta-analysis conducted by Prokopidis and colleagues in 2025 [41] reported no significant effect of WP supplementation on HOMA-IR in individuals under 50 years of age, whereas our findings indicate increased HOMA-IR in older adults, suggesting differential responses in insulin sensitivity across age groups. Generally, WP has been identified as a valuable nutritional strategy for improving lipid profiles, with particular benefits in reducing LDL, TCH, and TG, particularly when combined with regular exercise [79]. By contrast, a recent meta-analysis in adults aged <50 years reported reductions in LDL and TCH, particularly when WP was combined with exercise, and lower TG levels with [41].

In the field of gerontology, an inverse correlation between muscular strength and all-cause mortality, cardiovascular mortality, and the development of multiple chronic diseases has been shown [80]. Furthermore, the presence of frailty and sarcopenia, are significant contributors to cardiometabolic abnormalities, and risk of cardiovascular diseases [81], mediated by insulin resistance and chronic inflammation [82]. Our study shows the potential positive role of WP supplementation on lower-body muscular strength in older adults, that may be associated with greater functional capacity and higher quality of life [83]. This finding is at odds with the results of previous meta-analyses, which reported an absence of a significant beneficial effect on muscular strength, physical performance, or body composition in older adults [31,74]. Compared with Naclerio and Larumbe-Zabala (2016), who included resistance-trained adults aged 18 to 50 years, found that whey protein alone did not significantly improve either upper- or lower-body strength [84]. Meanwhile, our mixed results showed no significant change in upper-body strength but a significant increase in lower-body strength, suggesting potential age or training status-related differences in responsiveness to whey protein [84].

Overall, the present findings suggest that WP supplementation can enhance muscular strength in older adults without significantly altering body composition or cardiometabolic outcomes. Aging is associated with a decline in muscle strength, mass, and function, that increases the risk of frailty. Consequently, it may function as a preventative strategy to maintain functional capacity and reduce the risk of strength-related functional decline, particularly when combined with resistance exercise. From a clinical perspective, this finding suggests that protein supplementation may be a valuable strategy for maintaining functional strength and reducing the risk of falls, even in the absence of measurable muscle hypertrophy. Theoretically, the dissociation between strength gains and lean body mass highlights the role of neural adaptations and muscle quality in aging populations, consistent with the concept of anabolic resistance. The results of our study emphasize the necessity for integrated interventions, such as resistance training in conjunction with WP supplementation, to achieve more comprehensive enhancements in body composition and metabolic health. However, the efficacy of WP supplementation in enhancing body composition and cardiometabolic health-related indicators among aging populations remains to be elucidated. Consequently, future research should prioritize the development of standardized exercise protocols, the assessment of long-term safety, and the comparison of these findings with those observed for other protein sources. In addition, current data does not provide a satisfactory explanation for increases in fasting insulin and HOMA-IR following WP supplementation, and requires further research.

The present study has several limitations, which should be considered when interpreting the findings. First, it is imperative to consider the effects of increasing protein intake, particularly WP, on cardiovascular and metabolic health, despite the current lack of knowledge regarding the precise mechanisms involved [85]. Most included studies did not measure total protein intake, nor control the intake during the supplementation period. Second, we did not directly assess the effects of WP compared with other dietary proteins, which could be important as plant- and animal-based dietary proteins can exert differential effects on cardiometabolic risk factors [86]. Third, the use of vastly different dosages of WP in the included studies highlights the need to incorporate WP into individual diets according to current recommendations for older adults [87]. Fourth, the majority of trials did not control for total protein intake or baseline diet. This introduces a critical bias in the interpretation, which must be considered when analyzing the findings. Fifth, it is imperative to consider the potential for language bias, since we only included studies that were published in English, and the limited number of studies that reported on some of the cardiometabolic outcomes. Finally, the heterogeneity of the exercise modalities, the frequency of the exercise, and the intensity levels across the studies underscore the necessity for cautious interpretation of the results. In the future, it is imperative to ensure that the exercise protocols are more consistent.

## 5. Conclusions

The findings of the present study suggest that WP supplementation results in a moderate increase in lower-body muscle strength in older adults. However, the study did not demonstrate substantial benefits for body composition or cardiometabolic health markers. Conversely, increases in fasting insulin and HOMA-IR were shown, necessitating further research to ascertain the potential risks for this population. The present findings underscore the necessity of a meticulous examination of its metabolic effects and the continuation of long-term research. Subgroup analyses showed that WP with resistance training increased LBM but produced no other benefits, while WP alone improved lower-body strength. Further high-quality research is needed to elucidate these findings in order to optimize supplementation protocols, assess long-term outcomes, and confirm safety and effectiveness. These long-term randomized trials should also control for total protein intake and baseline diet.

## Figures and Tables

**Table 1:** Characteristics of participants and interventions.

Source, Year	Participants Characteristics					Intervention Characteristics		Intervention Characteristics
	Sample Size (Sex)	Health Status	Age (Years)	BMI (kg/m^2^)	Design	Duration	Type	
Amasene et al., 2019 [48]	28 (F and M)	Sarcopenia	WP + RT: 82.9 ± 5.59 PLA + RT: 81.7 ± 6.45	WP + RT: 27.4 ± 3.50 PLA + RT: 30.8 ± 6.53	RCT	12-wk	WP + Leucine	LBM, BFP, WHR, ST (Handgrip)
Arnarson et al., 2013 [49]	141 (F and M)	Apparently healthy	WP + RT: 73.3 ± 6.0 PLA + RT: 74.6 ± 5.8	WP + RT: 28.1 ± 4.4 PLA + RT: 29.4 ± 4.8	RCT	12-wk	WP	LBM, ST (Quadriceps—isometric)
Bach et al., 2022 [50]	31 (F and M)	Healthy	WP + RT: 66.9 ± 4.3 PLA + RT: 65.8 ± 5.0	WP + RT: 26.3 ± 2.2 PLA + RT: 25.4 ± 2.0	RCT	12-wk	WP	LBM, FM, ST (Peak torque—knee extension)
Chalé et al., 2013 [51]	80 (F and M)	Mobility-Limited	WP + RT: 78.0 ± 4.0 PLA + RT: 77.3 ± 3.9	WP + RT: 27.0 ± 3.2 PLA + RT: 26.9 ± 3.1	RCT	24-wk	WP	LBM, FM, ST (1-RM, leg press)
Dos santos et al., 2023 [63]	25 (F and M)	Chronic Heart failure	WP: 64.0 ± 4.9 PLA: 65.66 ± 17.2	WP: 28.6 ± 4.6 PLA: 26.8 ± 3.5	RCT	12-wk	WP	FBG, FM, BFP, WC, ST (Handgrip), TCH, TG, HDL, LDL
Dulac et al., 2021 [52]	40 (M)	Healthy	69 ± 7	WP + RT: 26.7 ± 3.0 PLA + RT: 25.4 ± 3.4	RCT	12-wk	WP	FBG, Insulin, HOMA-IR, BFP, FM, LBM, ST (Handgrip, isometric—knee extension)
Fernandes et al., 2018 [53]	32 (F)	Physically independent	WP + RT: 67.3 ± 4.1 PLA + RT: 67.8 ± 4.0	WP + RT: 25.9 ± 2.7 PLA + RT: 25.4 ± 2.6	RCT	12-wk	WP	FBG, FM, WC, WHR, TCH, TG, HDL, LDL
Griffen et al., 2022 [70]	36 (M)	Healthy	WP + RT: 68.0 ± 3.0 PLA + RT: 67.0 ± 3.0 WP: 66.0 ± 6.0 PLA: 67.0 ± 6.0	WP + RT: 26.6 ± 2.4 PLA + RT: 25.1 ± 2.7 WP: 25.0 ± 1.8 PLA: 25.1 ± 3.0	RCT	12-wk	WP + Leucine	Insulin, FM, BFP, WHR, WC, ST (1-RM, leg press)
HaB et al., 2022 [54]	40 (F and M)	Healthy	WP + RT: 71.5 ± 4.6 RT: 69.9 ± 4.5	WP + RT: 28.2 ± 2.3 RT: 26.9 ± 2.7	RCT	8-wk	WP + Leucine	WHR, ST (isometric—Knee extension)
Holwerda et al., 2018 [55]	41 (M)	Healthy	WP + RT: 69 ± 4.58 PLA + RT: 71 ± 4.47	WP + RT: 25.5 ± 2.74 PLA + RT: 25.1 ± 2.21	RCT	12-wk	WP + Leucine	LBM, FM, BFP, ST (1-RM—leg press)
Kirk et al., 2019 [61]	46 (F and M)	Healthy	WP + RT: 69 ± 6 RT: 66 ± 4	WP + RT: 27.4 ± 4.9 RT: 28.1 ± 7.4	RCT	16-wk	WP + Leucine	FBG, ST (1-RM—Leg press, chest press)
Kirk et al., 2020 [71]	100 (F and M)	Healthy	WP + RT: 69 ± 6 WP: 72 ± 6 CON: 68 ± 6 RT: 66 ± 4	WP + RT: 27.4 ± 4.9 WP: 27.1 ± 4.1 CON: 26.2 ± 4.5 RT: 28.1 ± 7.4	RCT	16-wk	WP + Leucine	FM, ST (Handgrip, maximum voluntary contraction—leg extension)
Kirk et al., 2021 [36]	73 (F and M)	Healthy	WP + RT: 68.59 ± 5.7 WP: 71.83 ± 6.5 CON: 68.16 ± 5.85 RT: 66.63 ± 3.92	27.06 ± 5.18	RCT	16-wk	WP + Leucine	TCH, HDL, LDL
Koopmans et al., 2024 [64]	43 (M)	Healthy	WP: 70.0 ± 5.0 PLA: 68.0 ± 5.0	WP: 24.4 ± 2.3 PLA: 23.8 ± 2.7	RCT	11-wk	WP	FM, ST (Handgrip)
Li et al., 2021 [65]	61 (F and M)	Healthy	WP: 71 ± 4 CON: 71 ± 4	WP: 21.8 ± 2.0 CON: 20.8 ± 2.2	RCT	24-wk	WP + Leucine	LBM, ST (Handgrip)
Mertz et al., 2021 [66]	78 (F and M)	Healthy	WP: 70.3 ± 4.3 PLA: 69.6 ± 3.9	WP: 25.2 ± 3.6 PLA: 26.0 ± 3.9	RCT	1 year	WP + Sucrose	LBM, BFP, ST (Peak torque—knee extension)
Mori et al., 2018 [56]	50 (F)	Healthy	WP + RT: 70.6 ± 4.2 RT: 70.6 ± 4.2	WP + RT: 22.1 ± 2.1 RT: 22.9 ± 2.9	RCT	24-wk	WP + Leucine	ST (Handgrip, knee extension)
Mori et al., 2022 [57]	46 (F and M)	Sarcopenia	WP + RT: 77.7 ± 3.3 RT: 77.6 ± 5.2	WP + RT: 20.3 ± 2.4 RT: 20.3 ± 2.9	RCT	24-wk	WP + Leucine	ST (Handgrip, knee extension)
Murphy et al., 2021 [67]	69 (F and M)	Low muscle mass	WP: 70 ± 5 PLA: 73 ± 7	WP: 24.8 ± 3.4 PLA: 25.4 ± 2.8	RCT	24-wk	WP + Leucine	FBG, Insulin, HOMA-IR, FM, ST (Handgrip, Isokinetic knee extension), TG, TCH, HDL, LDL
Nabuco et al., 2019 [58]	26 (F)	Sarcopenic obesity	WP + RT: 68.0 ± 4.2 PLA + RT: 70.1 ± 3.9	WP + RT: 26.4 ± 3.0 PLA + RT: 27.4 ± 3.0	RCT	12-wk	WP	FBG, Insulin, HOMA-IR, FM, BFP, WC, WHR, ST (1-RM—Knee extension, chest press), TG, TCH, LDL, HDL
Roschel et al., 2021 [60]	44 (F)	Pre-frail, frailty	72 ± 6	N R	RCT	16-wk	WP	LBM, BFP, ST (1-RM—leg press, chest press)
Soares et al., 2023 [62]	28 (M)	T2DM	WP + RT: 68.1 ± 4.5 PLA + RT: 68.9 ± 4.1	WP + RT: 29.3 ± 2.6 PLA + RT: 26.8 ± 3.8	RCT	12-wk	WP	FBG, Insulin, HOMA-IR, LBM, FM, ST (Handgrip)
Stojkovic et al., 2017 [68]	84 (F)	Postmenopausal women	WP: 68.9 ± 5.54 PLA: 69.3 ± 6.10	WP: 26.0 ± 3.69 PLA: 25.8 ± 4.06	RCT	18 months	WP	LBM, FM
Sugihara et al., 2018 [59]	31 (F)	Physically independent	WP + RT: 67.4 ± 4.1 PAL + RT: 67.8 ± 4.1	WP + RT: 25.6 ± 2.4 PLA + RT: 25.4 ± 2.6	RCT	12-wk	WP + Leucine	ST (1-RM—knee extension, chest press)
Zhu et al., 2015 [69]	181 (F)	Postmenopausal women	WP: 74.2 ± 2.8 PLA: 74.3 ± 2.6	WP: 26.1 ± 3.8 PLA: 27.2 ± 4.0	RCT	2 years	WP	ST (Handgrip, knee extension)

Abbreviations: WP: Whey Protein; RT: Resistance Training; PLA/CON: Placebo/Control; RCT: Randomized Controlled Trial; F: Female; M: Male; BMI: Body Mass Index; T2DM: Type 2 Diabetes Mellitus; LBM: Lean Body Mass; FM: Fat Mass; BFP: Body Fat Percentage; WC: Waist Circumference; WHR: Waist-to-Hip Ratio; ST: Strength; FBG: Fasting Blood Glucose; HOMA-IR: Homeostasis Model Assessment of Insulin Resistance; TCH: Total Cholesterol; TG: Triglycerides; HDL: High-Density Lipoprotein Cholesterol; LDL: Low-Density Lipoprotein Cholesterol; 1-RM: One-Repetition Maximum; wk: week(s).

## Data Availability

The original contributions presented in this study are included in the article/Supplementary Material. Further inquiries can be directed to the corresponding authors.

## Supplementary Materials

The following supporting information can be downloaded at: https://www.mdpi.com/article/10.3390/healthcare13212814/s1. Ref. [42] is cited in the supplementary material file.

## Abbreviations

The following abbreviations are used in this manuscript:

PRISMA	Preferred Reporting Items for Systematic Reviews and Meta-Analyses
PROSPERO	International Prospective Register of Systematic Reviews
PICOS	Population, Intervention, Comparator, Outcomes, Study design
WP	Whey Protein
PLA	Placebo
BF%	Body Fat Percentage
LBM	Lean Body Mass
WC	Waist Circumference
WHR	Waist-to-Hip Ratio
HOMA-IR	Homeostatic Model Assessment for Insulin Resistance
TG	Triglycerides
TCH	Total Cholesterol
LDL	Low-Density Lipoprotein Cholesterol
HDL	High-Density Lipoprotein Cholesterol
IQR	Interquartile Range
SD	Standard Deviation
PEDro	Physiotherapy Evidence Database
CMA3	Comprehensive Meta-Analysis version 3
WMD	Weighted Mean Difference
SMD	Standardized Mean Difference
CI	Confidence Interval
RCT	Randomized Controlled Trial
